# Viral Release Threshold in the Salivary Gland of Leafhopper Vector Mediates the Intermittent Transmission of Rice Dwarf Virus

**DOI:** 10.3389/fmicb.2021.639445

**Published:** 2021-02-04

**Authors:** Qian Chen, Yuyan Liu, Zhirun Long, Hengsong Yang, Taiyun Wei

**Affiliations:** Fujian Province Key Laboratory of Plant Virology, Institute of Plant Virology, Vector-borne Virus Research Center, Fujian Agriculture and Forestry University, Fuzhou, China

**Keywords:** intermittent transmission, rice dwarf virus, insect vectors, salivary glands, viral release threshold

## Abstract

Numerous piercing-sucking insects can persistently transmit viral pathogens in combination with saliva to plant phloem in an intermittent pattern. Insect vectors maintain viruliferous for life. However, the reason why insect vectors discontinuously transmit the virus remains unclear. Rice dwarf virus (RDV), a plant reovirus, was found to replicate and assemble the progeny virions in salivary gland cells of the leafhopper vector. We observed that the RDV virions moved into saliva-stored cavities in the salivary glands of leafhopper vectors via an exocytosis-like mechanism, facilitating the viral horizontal transmission to plant hosts during the feeding of leafhoppers. Interestingly, the levels of viral accumulation in the salivary glands of leafhoppers during the transmitting period were significantly lower than those of viruliferous individuals during the intermittent period. A putative viral release threshold, which was close to 1.79 × 10^4^ copies/μg RNA was proposed from the viral titers in the salivary glands of 52 leafhoppers during the intermittent period. Thus, the viral release threshold was hypothesized to mediate the intermittent release of RDV from the salivary gland cells of leafhoppers. We anticipate that viral release threshold-mediated intermittent transmission by insect vectors is the conserved strategy for the epidemic and persistence of vector-borne viruses in nature.

## Introduction

Several persistent plant viruses of agricultural importance are transmitted to plants by piercing–sucking insect vectors, such as leafhoppers, planthoppers, and whiteflies (Jia et al., 2018). These vectors are usually infected with viruses throughout their lifetime, yet they transmit the virus intermittently (Gamez, 1973; Reynaud and Peterschmitt, 1992; Muniyappa et al., 2000; Ammar and Nault, 2002; Pu et al., 2012). Intermittent transmission refers to the phenotype that after the latent period of persistent virus, insect vectors discontinuously transmit the virus for life, rather than transmitting it daily. The intermittent period ranges from 2 to 14 days, in which the insect vectors still remain viruliferous (Muniyappa et al., 2000; Pu et al., 2012). However, the reason why insect vectors intermittently transmit viruses and the association of intermittent transmission with viral infection in the plant hosts remain unknown. To address the mechanism that underlies the intermittent transmission of viruses by insect vectors, it is essential to understand how the viruses are released from the salivary glands of insect vectors.

In the salivary glands of insects, most salivary gland cells are filled with abundant apical plasmalemma-lined cavities in which saliva is stored (Mao et al., 2017). Virions are generally secreted together with saliva to the salivary cavities (Hogenhout et al., 2008; Wei and Li, 2016). The virions then successively move with saliva into a canal that leads to the salivary canal and a duct that allows virus-laden saliva to exit the stylets. The virions are ultimately ejected into the phloem of the plant, while insect vectors feed on susceptible hosts (Hogenhout et al., 2008; Wei and Li, 2016). Therefore, the salivary glands of insects serve as the last barrier for the circulation of the viral pathogen and determines whether an insect can transmit the virus. A better understanding of the mechanism that underlies how the virus overcomes the salivary glands barriers is essential to provide a basis for the control of viral diseases. Previous studies showed that the salivary gland release barrier referred to the apical plasmalemma that separates the salivary cavities for saliva storage (Gray and Gildow, 2003; Mao et al., 2017). Virus in the cytoplasm of salivary glands must pass through the apical plasmalemma and disseminate into the salivary cavities for transmission by insect vectors. Therefore, the transmission efficiency by insect vectors is correlated with the viral ability to overcome the salivary gland release barrier.

Rice dwarf virus (RDV), belonging to the *Phytoreovirus* genus of *Reoviridae* family, was the first plant virus discovered to be transmitted by insect vectors (Ishikawa, 1928; Attoui et al., 2012). RDV causes rice dwarf disease, which results in yield losses in southern China, Japan, and southeast Asia (Boccardo and Milne, 1984). This virus is primarily transmitted by the leafhopper vector *Nephotettix cincticeps* in a persistent-propagative manner, and the transmission process includes infective and non-infective periods (Ishikawa, 1928; Fukushi, 1940; Zhong et al., 2003). The virion is icosahedral and approximately 70 nm in diameter (Attoui et al., 2012). The viral genome possesses 12 double-stranded RNA (dsRNA) segments (S1-S12) that encode seven structural proteins (P1, P2, P3, P5, P7, P8, and P9) and five non-structural proteins (Pns4, Pns6, Pns10, Pns11, and Pns12) (Omura and Yan, 1999; Nakagawa et al., 2003; Miyazaki et al., 2010; Chen et al., 2015a). Once the virus gains access to the alimentary canal of leafhopper via the stylet and esophagus, the RDV virions specifically recognize and bind to the undefined receptors of the filter chamber and enter the epithelium (Chen et al., 2011). The viral life cycle begins when the viroplasm, which is composed of the matrix aggregated by non-structural proteins Pns6, Pns11, and Pns12, is generated for viral propagation (Wei et al., 2006b; Chen et al., 2015b). At approximately 12 days post first access to diseased plants (padp), the RDV reaches the salivary glands of the most viruliferous leafhoppers (Chen et al., 2011). The leafhopper then becomes RDV transmittable at approximately 14 days padp (Honda et al., 2007; Chen et al., 2011). However, how the leafhopper transmits RDV and how RDV releases from the salivary glands and infects plants via the stylets of the leafhopper vector remain unknown.

To understand how a virus releases from the salivary gland and infects plant hosts, the system of RDV–leafhopper system was investigated. In this study, by applying immunofluorescence and electron microscopy analyses, RDV infection, replication in, and release from the salivary gland cells of leafhopper vectors were characterized. The properties of viral intermittent transmission by leafhoppers were also shown. Finally, we proposed a putative threshold for viral release to interpret the phenotype of viral intermittent transmission by the leafhopper vectors. We hypothesized that this viral intermittent release threshold mediates the viral release from leafhopper vectors for the effective infection of the virus into the plant host.

## Materials and Methods

### Insects, Viruses, and Antibodies

Uninfected *N. cincticeps* leafhopper individuals were collected from Yunnan Province, southwest China and propagated for several generations at 25 ± 3°C in the laboratory. Rice samples infected with RDV were also initially collected from Yunnan Province. These diseased plants served as an original viral source for transmission by *N. cincticeps* to rice plants (*Oryza sativa* L. ssp. *Japonica*, variety *Nipponbare*) under greenhouse conditions. The infected rice plants were tested using RT-PCR and PCR assays to make sure that the rice plants were singly infected with RDV in the absence of rice orange leaf phytoplasma.

Rabbit polyclonal antisera specific for RDV and Pns6 were provided by Dr. Toshihiro Omura (National Agricultural Research Center, Japan). IgGs of RDV and Pns6 were directly conjugated to fluorescein isothiocyanate (FITC) and rhodamine, respectively, according to manufacturer’s instructions (Thermo Fisher Scientific, United States). The virus-FITC and Pns6-rhodamine were used for immunofluorescence detection. Actin dye rhodamine-phalloidin was obtained from Thermo Fisher Scientific (Waltham, MA, United States). The antibodies against the glyceraldehyde-3-phosphate dehydrogenase (GADPH) were obtained from Sangon Biotech (Shanghai, China).

### Virus Acquisition and Transmission

Uninfected second-instar nymphs were first allowed to feed on rice plants with RDV for 2 days and then transferred to healthy plants for 12 days. At 14 days padp, the salivary glands were dissected for immunofluorescence assays or electron microscopy.

To characterize the profile of RDV transmission by a group of leafhoppers, the leafhoppers that fed on diseased rice plants were then sequentially kept on healthy rice seedlings for 10 days and individually fed on a healthy rice seedling in one glass tube for 24 h (**Supplementary Figure 1**). The leafhoppers were then transferred daily to new healthy rice seedlings for 13 days (**Supplementary Figure 1**). All of the rice seedlings tested were planted in an insect-proof greenhouse for 60 days (**Supplementary Figure 1**). To determine whether RDV was transmitted by leafhoppers to plants, RT-PCR was performed to test the presence/absence of the RDV P8 gene, which encodes a major outer capsid protein, in the tested plants.

To determine the viral genome copies in the salivary glands of viruliferous leafhoppers in the transmitting and intermittent periods at 19 days padp, leafhoppers that had previously fed on diseased plants for 2 days were individually fed on a healthy rice seedling in a glass tube for 24 h. The bodies of tested leafhoppers were then collected for RT-PCR to determine whether or not the insects were infected. The tested rice seedlings were grown for 60 days and then examined using RT-PCR for the presence of the RDV P8 gene to determine whether or not RDV was transmitted by leafhoppers, ultimately enabling the transmitting or non-transmitting leafhoppers to be distinguished from the population tested. RT-qPCR was then conducted on the corresponding salivary glands of transmitting or non-transmitting leafhoppers to determine the viral gene copies of the major outer capsid protein P8.

To visualize the viral accumulation in salivary glands of leafhoppers during the transmitting or intermittent periods, the salivary glands of the leafhoppers tested post 24 h inoculation feeding were dissected for immunofluorescence assays. The methods of viral acquisition, transmission, and distinguishment for transmitting or non-transmitting viruliferous leafhoppers from the population tested were the same as those described above.

### RNA Extraction and RT-qPCR Detection

To extract the total RNA of individual insect bodies, the individual insects were collected in one 1.5 mL Eppendorf tube. The tubes were then placed in liquid nitrogen. The insect bodies were ground with pestles and then lysed with 200 μL TRIzol reagent (Thermo Fisher Scientific). To extract the total RNA of salivary glands of each individual insect, a pair of salivary glands was dissected and collected in one 0.5 mL tube. The salivary glands were then lysed with 100 μL TRIzol reagent. The subsequent procedures were conducted following the manufacturer’s instructions. The final concentration of RNAs was determined using a NanoDrop 1000 (Thermo Fisher Scientific).

To absolutely quantify the viral titers in the salivary glands of infected leafhoppers, a standard curve of RDV P8 was first established. In brief, the concentration of the plasmid DNA, including the RDV P8 gene, was determined using the NanoDrop 1000. A 10-fold dilution series of plasmid was then prepared in RNase-free water, and the copy number of the RDV P8 gene was calculated using the following formula: (DNA amount × 6.022 × 10^23^)/(plasmid length × 1 × 10^9^ × 650). The diluted plasmids were analyzed for their Ct value using qPCR assays with specific primers of RDV P8 (forward primer 5′-tacagccatcagctaagccaaa-3′) and reverse primer 5′-ccgcaacagaccgaaaca-3′). The qPCR assays were performed in a Mastercycler Realplex4 real-time PCR system (Eppendorf, Hamburg, Germany) using GoTaq qPCR Master Mix kit (Promega, Madison, WI). Based on the correlation of the logarithm of plasmid copy number to base 10 with the corresponding Ct value, the equation y = –3.506x + 42.981 (x is the logarithm of plasmid copy number to base 10, y is the Ct value, and *R*^2^ = 0.9987) was established.

The viral titers in the salivary glands of infected leafhoppers were then determined. The first-strands cDNA of RDV P8 derived from the RNAs of salivary glands were synthesized using the specific primer (forward primer 5′-tacagccatcagctaagccaaa-3′) and then analyzed for their Ct values using qPCR assays. The system and program of the qPCR assay for cDNA were the same as those of the qPCR assay for plasmid DNA. Based on the Ct value of each cDNA and the equation y = –3.506x + 42.981, the copy number of RDV P8, which was used to judge for viral genome copy, was determined as the log of the copy number per microgram of insect RNA.

### Immunofluorescence Assay

To characterize the viral infection in and release from salivary glands, uninfected second-instar nymphs were allowed to feed on rice plants infected with RDV for 2 days and were then kept on healthy rice seedlings. At 12 days padp, the salivary glands of these tested leafhoppers were dissected, fixed in 4% paraformaldehyde in PBS for 10 h, and then permeabilized in 0.2% Triton X-100 for 24 h. Following previously described methods (Chen et al., 2017), the salivary glands were ultimately immunolabeled with antibodies against virus-FITC for RDV particles, Pns6-rhodamine for viroplasm, or rhodamine-phalloidin for actin. The resulting samples were observed using a Leica TCS SP5 inverted confocal microscope (Wetzlar, Germany).

### Electron Microscopy

The salivary glands dissected from infected *N. cincticeps* were fixed, dehydrated, and embedded. Ultrathin sections were then cut as previously described (Mao et al., 2017).

### Statistical Analyses

All data for viral titers in the salivary glands of leafhoppers were analyzed using a two-tailed *t*-test in GraphPad Prism 6 (San Diego, CA, United States).

## Results

### RDV Infection and Replication in Salivary Gland Cells

**FIGURE 1:**
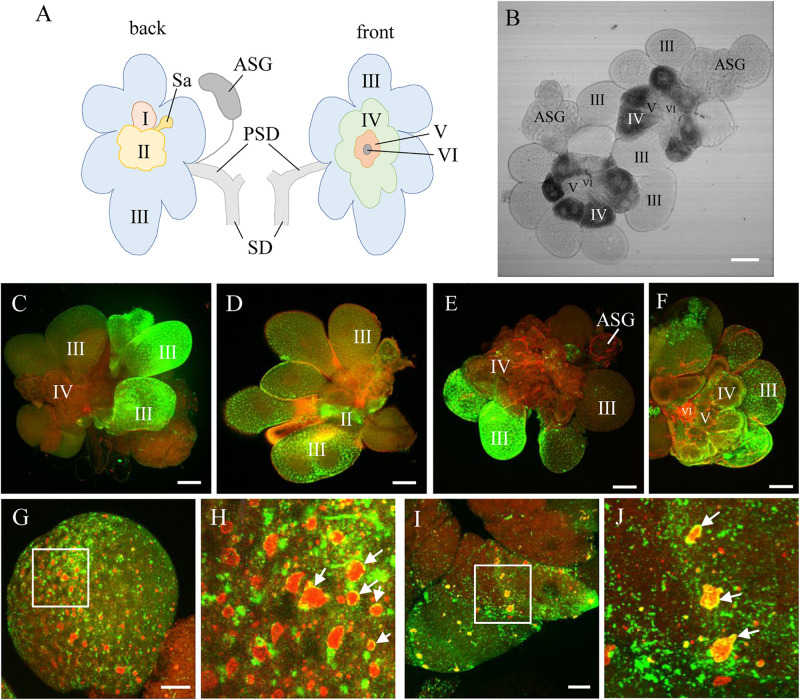
RDV infection and replication in the salivary gland cells of viruliferous leafhoppers. **(A)** Schematic illustration of a salivary gland from an *N. cincticeps* leafhopper, modified from Sogawa (1965). **(B)** Transmission light micrograph of one pair of salivary glands from an *N. cincticeps* leafhopper. The principal salivary gland of *N. cincticeps* consists of six types of cells (I to IV). **(C–F)** Immunofluorescence microscopy showing RDV infection in different types of salivary gland cells. **(G,H)** RDV replication in type III- **(G,H)** and IV- **(I,J)** cells. Salivary glands of leafhoppers at 12 days padp were stained with virus-FITC (green), actin dye phalloidin-rhodamine (red) **(C–F)**, or Pns6-rhodamine (red) **(G,H)**, and then examined by confocal microscopy. The arrows indicate viroplasm. Panels **(H,J)** are enlarged images of the boxed areas in panels **(G,I)**, respectively. PSD, principal salivary duct; SD, salivary duct; Sa, spherical appendage; ASG, accessory salivary gland. Bars, 100 μm **(B–F)**, 20 μm **(G,I)**.

To address how RDV was released from the cells of salivary gland to overcome the salivary gland release barrier, the accumulation of RDV in the salivary glands was first studied. The salivary glands of *N. cincticeps* consist of a pair of principal and accessory salivary glands. The principal gland contains six types of cells (I–VI) (Figures 1A,B). The type III-cell was the largest and separated from each other, while the type VI-cells situated at the center of the type V-cells were the smallest. Immunofluorescence assays showed that at 12 days padp, RDV antigens were localized to the type III-cells (Figure 1C); type II- and III-cells (Figure 1D); type III-cells, type IV-cells, and accessory salivary glands (Figure 1E), or they were found throughout the principal salivary glands (Figure 1F). These results indicated the high frequency of RDV infection in the type III-cells.

The antibodies against the viral non-structural protein Pns6, which is a component of the viroplasm matrix (Wei et al., 2006b; Chen et al., 2015b), were used to examine viral replication. Immunofluorescence assays showed that various amounts of viroplasm were distributed in each type of cell, such as type III- and IV-cells (Figures 1G–J). As indicated by the yellow in Figures 1G–J, RDV was clearly localized at the periphery of viroplasm. These results indicated that RDV could replicate more progeny virions in most cells of the salivary gland.

### RDV Release From Salivary Gland Cells

**FIGURE 2:**
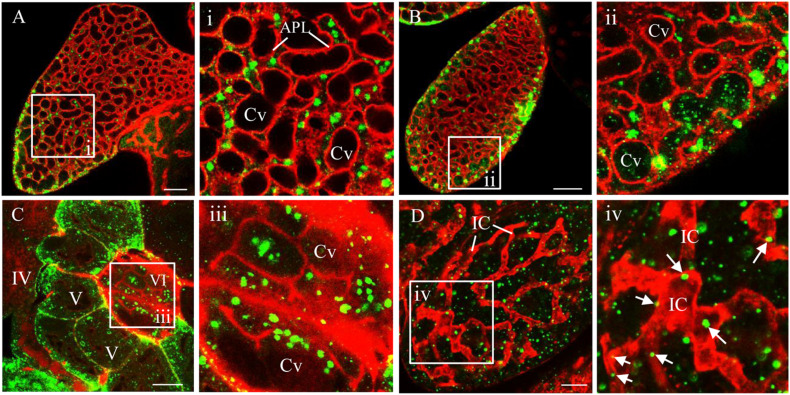
Immunofluorescence microscopy showing the release of RDV from salivary gland cells. **(A)** Abundant RDV virions that have accumulated inside the cytoplasm at the periphery of apical plasmalemma of type III-cells of salivary gland. **(B–D)** A number of RDV virions entering the cavities of type III **(B)** or VI **(C)** cells or into the canaliculi of type IV-cells of salivary gland **(D)**. The salivary glands of leafhoppers at 12 days padp were stained with virus-FITC (green) or actin dye phalloidin-rhodamine (red), and then examined by confocal microscopy. Arrows indicate virus in the canaliculi. Panels i to iv are enlarged images of the boxed areas in panels **(A–D)**, respectively. APL, apical plasmalemma; Cv, cavity; IC, intracellular canaliculi. Bars, 20 μm **(A–C)**, 10 μm **(D)**.

Using immunofluorescence assays, we then investigated the process of RDV release from salivary gland cells into salivary cavities. The type III-cells were filled with apical plasmalemma-lined saliva-storing cavities, which formed the loose network establishing the intracellular space of secretory cells (Figure 2A). At 12 days padp, the RDV virions appeared as discrete, punctate inclusions inside or at the periphery of salivary cavities in type III- and VI-cells (Figures 2A–C). In type IV-cells, intracellular canaliculi, which discharged the main protein components for the sheath saliva (Sogawa, 1967), were immunostained with the actin dye phalloidin-rhodamine and penetrated the cytoplasm in a coarse reticular manner (Figure 2D). A large number of virions were scattered throughout the cytoplasm of type IV-cells or located within the canaliculi (Figure 2D). Together, these results provide evidence for the viral release from salivary gland cells via entry into the cavities or intracellular canaliculi.

**FIGURE 3:**
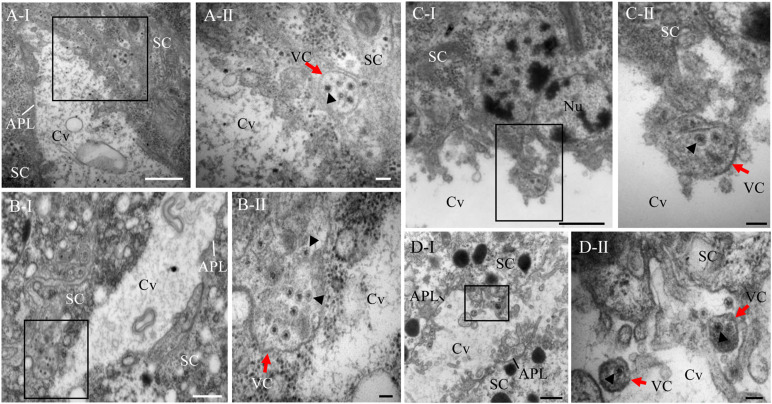
Electron micrographs demonstrating the entry of RDV into cavities by utilizing vesicular compartments via an exocytosis-like process. **(A)** Virus-laden vesicular compartments within the cytoplasm of salivary gland cells. **(B)** Virus-laden vesicular compartments in the cytoplasm moving close to the salivary cavities. **(C)** Virus-laden vesicular compartments enter into the salivary cavities by fusing with the apical plasmalemma. **(D)** Virus-laden vesicular compartments interspersing within the salivary cavities. Red arrows indicate virus-laden vesicular compartments. Black arrow heads indicate the virions in vesicular compartments. Panels II are enlarged images of the boxed areas in panels I of **(A–D)**, respectively. SC, salivary cytoplasm; APL, apical plasmalemma; Cv, cavity; VC, vesicular compartment. Bars, 500 nm **(A-I,B-I,C-I,D-I)**, and 100 nm **(A-II,B-II,C-II,D-II)**.

In addition, electron microscopy showed that double-layered RDV particles of approximately 70 nm in diameter were sequestered within vesicular compartments in various sizes in the cytoplasm of salivary gland cells or at the periphery of salivary cavities (Figures 3A,B). The migration of virus-laden vesicular compartments that were close to the salivary cavities led to the fusion of the apical plasmalemma with the vesicular compartments (Figure 3C). Finally, virus-laden vesicular compartments were released to the salivary cavities (Figure 3D). These observations indicate that the release of RDV virions into the saliva-stored cavities takes advantage of vesicular compartments via an exocytosis-like manner.

### Intermittent Transmission of RDV by *N. cincticeps*

**FIGURE 4:**
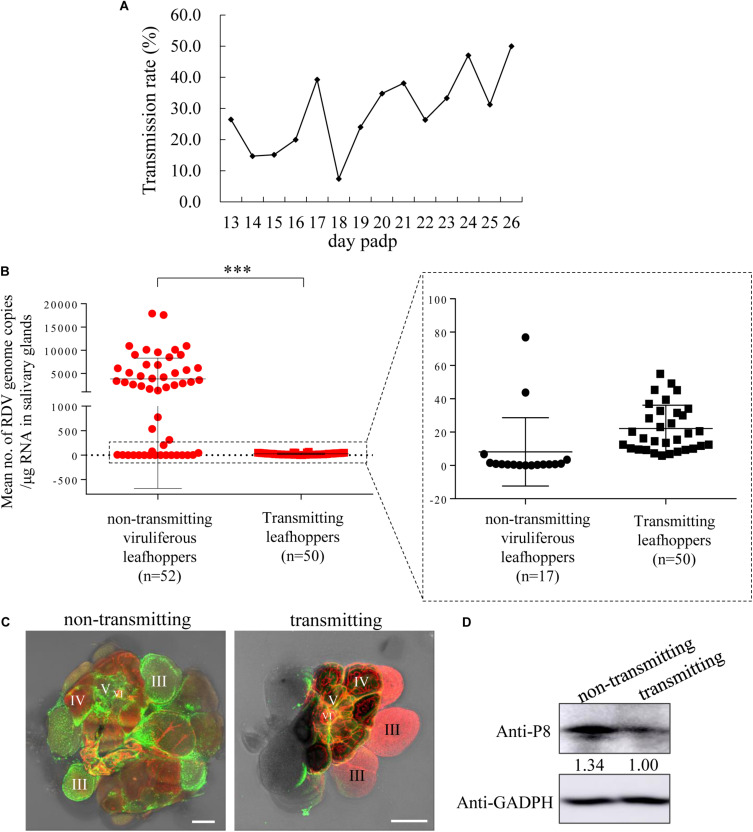
RDV titers in the salivary glands of leafhoppers during the transmitting period were significantly lower than those in the salivary glands of viruliferous leafhoppers during the intermittent period. **(A)** The profile of transmission rate of RDV by a group of leafhoppers per day. The transmission rate was determined using RT-PCR to detect the presence of RDV P8 gene in plants. **(B)** Viral titers in the salivary glands of transmitting and viruliferous leafhoppers during the intermittent period. An RT-qPCR assay of viral titers in the salivary glands of transmitting leafhoppers was highly significantly lower than that of the non-transmitting viruliferous leafhoppers at 19 days padp. *⁣*⁣*P < 0.0001. **(C)** Immunofluorescence microscopy exhibiting low viral infection in the salivary glands of the transmitting leafhoppers compared with viruliferous leafhoppers during the intermittent period. **(D)** Western blot of RDV P8 and GADPH in the salivary glands of transmitting leafhoppers and viruliferous leafhoppers during the intermittent period. The samples were separated by SDS-PAGE and the presence of P8 was examined with P8-specific antibodies. The relative intensities of bands in the analyses of P8 are shown below the bands. GADPH detected with GADPH-specific IgG is shown to demonstrate the loading of equal amounts of protein.

To understand the effect of RDV release via virus-laden vesicular compartments on viral transmission, the profiles of RDV transmission by *N. cincticeps* from 13 to 26 days padp was characterized. The curve for the daily transmission rates of RDV by individual *N. cincticeps* showed a wave pattern rather than a stable trend (Figure 4A), revealing a viral intermittent transmission pattern (Table 1). Approximately 61.8% of individual *N. cincticeps* showed the following pattern: they transmitted RDV for 1 day, then ceased transmission, and then some days later transmitted the virus for 1 day. The period of intermittent transmission ranged from 1 to 13 days (Table 2). Approximately 38.2% of *N. cincticeps* displayed the ability to continuously transmit RDV, and the period of transmission ranged from 2 to 5 days. The intermittence of viral transmission by individual *N. cincticeps* clarified the wave pattern observed in the graph of the daily transmission rate (Figure 4A).

**TABLE 1:** Pattern of daily transmission of RDV by individual leafhoppers.

Insect no.	Day padp													
	13	14	15	16	17	18	19	20	21	22	23	24	25	26
1	∘	•	∘	∘	∘	∘	∘	∘	D					
2	∘	∘	∘	∘	∘	∘	∘	∘	∘	∘	∘	•	•	•
3	•	∘	∘	•	•	∘	•	•	•	•	•	D		
4	•	∘	D											
5	∘	∘	∘	∘	∘	∘	∘	∘	•	∘	•	•	•	•
6	∘	∘	∘	•	•	•	∘	∘	•	∘	D			
7	•	∘	•	∘	∘	∘	∘	•	∘	∘	•	•	•	•
8	•	∘	•	∘	∘	∘	D							
9	∘	∘	∘	∘	∘	∘	∘	•	∘	∘	∘	∘	∘	∘
10	∘	∘	∘	∘	∘	∘	∘	∘	∘	∘	•	•	D	
11	•	∘	∘	∘	∘	∘	∘	∘	∘	∘	∘	∘	∘	∘
12	∘	∘	•	•	∘	D								
13	∘	•	∘	∘	∘	∘	∘	•	•	∘	∘	∘	∘	•
14	∘	∘	∘	∘	∘	∘	•	•	•	D				
15	•	∘	∘	∘	D									
16	∘	∘	∘	∘	∘	∘	∘	∘	∘	∘	∘	∘	∘	•
17	•	∘	D											
18	∘	∘	•	∘	•	∘	•	∘	∘	∘	∘	∘	∘	∘
19	∘	∘	∘	∘	∘	•	•	•	∘	•	∘	•	•	•
20	∘	•	∘	D										
21	•	∘	∘	•	∘	∘	∘	∘	∘	∘	∘	∘	∘	∘
22	∘	∘	∘	∘	•	∘	•	∘	•	D				
23	∘	∘	•	∘	D									
24	∘	∘	∘	∘	•	∘	∘	∘	•	∘	∘	∘	∘	∘
25	∘	∘	∘	∘	•	∘	∘	•	D					
26	∘	∘	∘	∘	•	∘	∘	∘	•	•	•	•	∘	•
27	∘	∘	∘	∘	∘	∘	∘	∘	∘	•	∘	•	∘	∘
28	•	∘	∘	D										
29	∘	•	∘	•	•	∘	•	D						
30	∘	∘	∘	∘	•	∘	∘	∘	∘	∘	∘	∘	∘	∘
31	∘	•	∘	D										
32	∘	∘	∘	•	∘	∘	∘	•	∘	•	•	•	•	•
33	∘	∘	∘	∘	•	∘	D							
34	∘	∘	∘	∘	•	∘	∘	D						

**∘, tested plant was not infected; •, tested plant was infected; D, the insect died.**

**TABLE 2:** Viral genome copies in the salivary glands of viruliferous leafhoppers during the intermittent period.

Insect no.	Copies/μg RNA
1	1.09E + 04
2	2.84E + 03
3	6.88E + 03
4	1.62E + 03
5	1.96E + 03
6	2.46E + 03
7	5.06E + 03
8	1.20E + 01
9	4.02E + 01
10	5.35E + 02
11	2.26E + 03
12	1.01E + 04
13	8.98E + 03
14	6.10E + 03
15	7.75E + 02
16	1.03E + 00
17	8.49E + 03
18	3.13E + 03
19	1.76E + 04
20	4.42E + 03
21	5.12E + 03
22	4.15E + 03
23	6.79E + 03
24	1.01E + 04
25	2.59E + 03
26	6.36E + 00
27	1.09E + 04
28	1.56E + 00
29	4.38E + 01
30	5.71E + 03
31	8.52E + 00
32	8.98E + 03
33	3.88E + 03
34	3.35E + 03
35	9.55E + 03
36	6.15E + 03
37	3.11E + 02
38	3.55E + 03
39	1.28E + 03
40	7.34E + 00
41	3.46E + 00
42	1.79E + 04
43	5.55E + 00
44	2.04E + 02
45	5.11E + 00
46	7.68E + 01
47	3.84E + 00
48	6.76E + 00
49	6.56E + 00
50	7.27E + 00
51	9.77E + 00
52	3.07E + 03

### A Release Threshold Mediating Viral Intermittent Release

To address the reason for viral intermittent transmission by *N. cincticeps* leafhoppers, the viral titers in salivary glands of *N. cincticeps* during the intermittent or transmitting periods were analyzed. The gene copy number of RDV P8 in the salivary glands of *N. cincticeps* served as viral titers. The results showed that the total RNA amount of a pair of salivary glands of one adult leafhopper ranged from 822 to 1,524 ng. The total RNA amount of one adult leafhopper ranged from 2,145 to 9,769 ng. At 19 days padp, the mean copy number of the viral genome in the salivary glands of 50 transmitting adult leafhoppers was 2.76 × 10^1^ copies/μg RNA, which was significantly lower than the mean copy number of 3.8 × 10^3^ copies/μg RNA in the salivary glands of 52 leafhoppers during the intermittent period (Figure 4B). The immunofluorescence assays showed that the RDV antigens in the salivary glands of leafhoppers during the transmitting period were less than those of the leafhoppers during the intermittent period (Figure 4C). Western blots also indicated a lower accumulation of RDV P8 in the salivary glands of *N. cincticeps* vectors during the transmitting period compared with those of viruliferous leafhoppers during the intermittent period (Figure 4D). These findings indicated that the viral load in the salivary glands decreased significantly owing to viral release into the rice plants, which was also the process of viral transmission by leafhoppers. Moreover, the transmitting and non-transmitting behaviors of viruliferous leafhoppers could result from a release threshold for viral release.

We then analyzed the number of viral genome copies in the salivary glands of 52 leafhoppers in the intermittent period (copy number ranging from 1.03 × 10^0^ to 1.79 × 10^4^ copies/μg RNA; Table 2) and deduced that the highest 1.79 × 10^4^ copies/μg RNA were very close to the viral release threshold. Below this putative RDV release threshold, the RDV tended to accumulate, and few of the virions could release from the salivary gland cells in most leafhoppers (Table 2). Once the viral accumulation in the salivary glands of leafhoppers increased above the viral release threshold, the virus was released from the salivary gland cells and entered the salivary cavities for transmission by most leafhoppers. Therefore, this release threshold possibly mediated the intermittent transmission of RDV by *N. cincticeps*.

## Discussion

To date, few reports have clarified the mechanism by which viruses overcome the salivary glands release barrier. Non-propagative persistent luteoviruses and propagative rhabdoviruses can overcome this barrier via transcytosis or membrane budding (Gildow, 1982;Brault et al., 2006; Mao et al., 2017). The propagative rice gall dwarf virus(RGDV) utilizes virus-induced inclusion as a vehicle and causes an exocytosis-like process for viral release into the salivary cavities (Mao et al., 2017). In this study, we found that the progeny RDV could release from salivary gland cells and enter the salivary cavities utilizing virus-laden vesicular compartments via the exocytosis-like process (Figures 1–3). These viral releases caused intermittent transmission of RDV by most leafhopper vectors (Table 1). Most importantly, the viral titers in the salivary glands of leafhoppers, which were in the transmitting period, were significantly lower than the viral titers in the salivary glands of leafhoppers, which were in the intermittent period (Figure 4). We proposed that the putative RDV release threshold (close to 1.79 × 10^4^ copies/μg RNA) (Table 2) mediated the viral intermittent transmission by leafhoppers, thus, facilitating RDV effective infection in the plant host.

### Salivary Gland Release Barrier

The salivary gland of leafhoppers secretes two different types of saliva, namely, coagulable and watery saliva (Sogawa, 1968). At the beginning of feeding, leafhoppers eject coagulable saliva to form salivary sheaths that surround the stylets for protection (Hattori et al., 2005). The watery saliva, which lubricates the stylet and introduces enzymes, ejects when the stylet penetrates the host plant tissues (Hattori et al., 2005). Simultaneously, virus is introduced with the watery saliva into the plant. Type III-cells secrete watery saliva; type IV-cells secrete the components of coagulable saliva, and type V-cells primarily secrete phenolase (Sogawa, 1967, 1968). We found that the virus passed through the apical plasmalemma of type III- and VI-cells, which then accessed the salivary cavities and mixed with watery saliva for release. In type IV-cells, the RDV entered the intracellular canaliculi and mixed with coagulable saliva for release. However, the biological significance of virus introduction with coagulable saliva that would solidify into the salivary sheath was unknown. However, we hypothesized that the main pathway of viral entry into plants hosts is the viral release from type III-cells and introduction into watery salivary, which is owing to the highest number of type III-cells and well liquidity and the high volume of watery saliva. Therefore, most viral release is associated with the apical plasmalemma of type III-cells, and the intermittent release from salivary gland cells may be primarily controlled in type III-cells.

Because the viral replication, accumulation, and spread in insect vectors are persistent, it was thought that salivary glands acted as the key barriers that affected viral intermittent release from salivary gland cells, leading to viral intermittent transmission. Salivary glands acted as a viral reservoir. If the viral release was simultaneous with viral replication, the viral titers would be stable, and the viral titers would be non-distinctive among the salivary glands of leafhoppers. However, we found that the viral titers within the salivary glands of leafhoppers during the transmitting period was significantly lower than the viral titers within the salivary glands of leafhoppers during the intermittent period. Moreover, in the group of viruliferous leafhoppers during the intermittent period, the viral titers in salivary glands were not consistent, and different individuals had large variations in viral titers (Table 2). Therefore, it was presumed that a certain threshold of viral release likely existed during a period of viral accumulation.

### Viral Release Threshold

**FIGURE 5:**
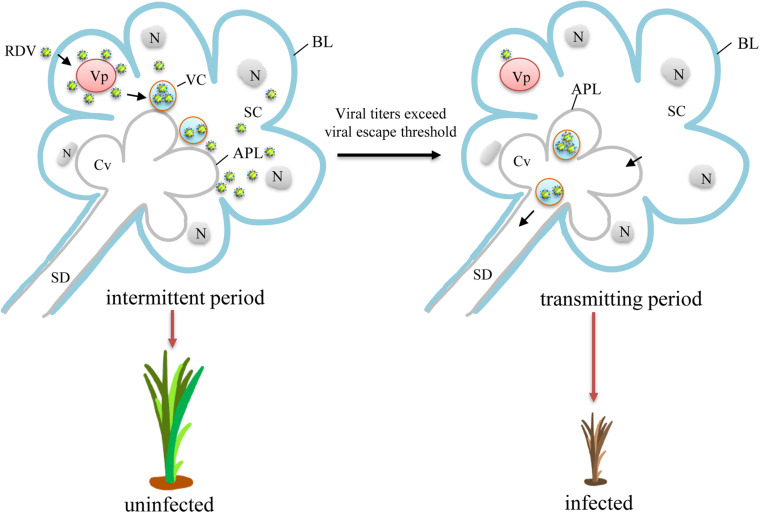
A proposed model for viral intermittent transmission controlled by the viral release threshold. Viruses first access the cytoplasm of salivary glands and begin replication for the production of progeny virions. Abundant new virions are then formed and sequestered in vesicular compartments before their final release to salivary cavities in an exocytosis-like manner. During this process, the level of viral accumulation is still below the virus release threshold, and the leafhopper does not transmit the virus. Therefore, intermittent periods of viral transmission are observed. With the continued replication, the level of viral accumulation gradually exceeds the virus release threshold. Thus, the newly formed virions traverse the apical plasmalemma and enter the salivary cavities in a free form or using virus-laden vesicular compartments. The virions then enter the salivary duct. Once the insects feed or probe, virions are ejected with saliva into the phloem of plant hosts, causing viral infection and transmission. Vp, viroplasm; APL, apical plasmalemma; BL, basal lamina; Cv, cavity; N, nucleus; SC, salivary cytoplasm; SD, salivary duct; VC, vesicular compartment.

Among the viral titers in salivary glands of viruliferous leafhoppers during the intermittent period, the highest viral titer was likely to be close to the viral release threshold. A model for viral intermittent transmission controlled by viral release threshold was then proposed (Figure 5). Viruses in the salivary glands of viruliferous leafhoppers during the intermittent period are still accumulating (Figure 5). Once the level of viral accumulation exceeds the threshold, virions are released from the salivary gland cells and infect the plant hosts. We consider this to be the threshold-controlled viral release strategy. When the level of viral accumulation decreases below the virus release threshold, leafhoppers enter the intermittent period (Figure 5). Once the level of viral accumulation reaches or exceeds the virus release threshold, the leafhopper once again transmits the virus (Figure 5).

An example of a viral release threshold that controls the release of plant viruses has previously described to have taken place during the infection of Southern rice black-streaked dwarf virus (SRBSDV) in planthoppers (Lan et al., 2016). The reason why the SRBSDV could infect but not be transmitted by the incompetent vectors is that the viral titers in the midgut epithelium are unable to reach the threshold needed for viral dissemination into the midgut muscles and then into the salivary glands (Lan et al., 2016). This suggests that the viral release threshold plays an important and universal role in mediating the viral ability to overcome barriers in insect vectors.

### Viral Intermittent Transmission

The intermittent transmission of insect-borne plant viruses has been shown to occur in leafhoppers, planthoppers, and whiteflies (Gamez, 1973; Reynaud and Peterschmitt, 1992; Muniyappa et al., 2000; Ammar and Nault, 2002; Pu et al., 2012). However, the mechanism that underlies the intermittent transmission remains unknown. Among rice-infecting viruses, the SRBSDV, which over the past decade has caused the severe loss of rice production throughout southern China and northern Vietnam, has been recorded to be continuously transmitted by planthopper vectors (Pu et al., 2012). The longest period of continuous transmission of SRBSDV could be up to 22 days (Pu et al., 2012), while we found that the period of continuous transmission for RDV was only 5 days. Therefore, the longer period of continuous transmission of SRBSDV compared with that of RDV may be one of the reasons why SRBSDV has caused greater damage to rice production than RDV.

What is the biological significance of viral intermittent transmission by insect vector? We hypothesize that there are two points at least. (1) It was presumed that the intermittent transmission correlated with the viral replication cycle, because in the salivary gland cells, the period from the RDV replication to the assembly of progeny virions, together with the induction of various inclusion bodies formation, such as vesicles, requires at least one cycle of viral replication (24 h) (Wei et al., 2006a,b; Chen et al., 2012). (2) It is known that to establish an infection in a plant host, a certain viral titer (dilution limit point) is required. The virus, which accumulated to some degree, guaranteed the successful and effective infection of the virus into the plant host after release. From these perspectives, viral intermittent transmission and the existence of virus release threshold are reasonable. We anticipate that the viral release threshold-mediated intermittent transmission by insect vectors is the conserved strategy for the epidemic and persistence of vector-borne viruses in nature.

## Data Availability Statement

All datasets generated for this study are included in the article/supplementary material, further inquiries can be directed to the corresponding author/s.

## Author Contributions

QC and TW designed the experiments, wrote and revised the manuscript. YL conducted the transmission electron microscopy experiments. QC, ZL, and HY collected the samples and conducted the biological experiments. QC conducted the immunofluorescence assay. All authors contributed to the article and approved the submitted version.

## Conflict of Interest

The authors declare that the research was conducted in the absence of any commercial or financial relationships that could be construed as a potential conflict of interest.

## References

[B1] Ammar E. Nault L. R. (2002). Virus transmission by leafhoppers, planthoppers and treehoppers (auchenorrhyncha, homoptera). *Adv*. *Bot*. *Res*. 36 141–167. 10.1016/S0065-2296(02)36062-2

[B2] Attoui H. Mertens P. P. C. Becnel J. Belaganahalli S. Bergoin M. Brussaard C. P. (2012). “Family reoviridae,” in *Virus Taxonomy: Ninth Report of theInternational Committee for the Taxonomy of Viruses*, eds King A. M. Q. Adams M. J. Carstens E. B. Lefkowits E. J. (New York, NY: Elsevier), 541–637. 10.1007/BF01309873

[B3] Boccardo G. Milne R. G. (1984). “Plant reovirus group,” in *CMI/AAB Descriptions of Plant Viruses, no. 294*, eds Morant A. F. Harrison B. D. (Kew: Commonwealth Microbiology Institute/Association of Applied Biology), 294.

[B4] Brault V. Herrbach E. Reinbold C. (2006). Electron microscopy studies on luteovirid transmission by aphids. *Micron* 38 302–312. 10.1016/j.micron.2006.04.005 16750376

[B5] Chen H. Chen Q. Omura T. Uehara-Ichiki T. Wei T. (2011). Sequential infection of rice dwarf virus in the internal organs of its insect vector after ingestion of virus. *Virus Res*. 160 389–394. 10.1016/j.virusres.2011.04.028 21570430

[B6] Chen Q. Chen H. Jia D. Mao Q. Xie L. Wei T. (2015b). Nonstructural protein Pns12 of Rice dwarf virus is a principal regulator for viral replication and infection in its insect vector. *Virus Res*. 210 54–61. 10.1016/j.virusres.2015.07.012 26200955

[B7] Chen Q. Chen H. Mao Q. Liu Q. Shimizu T. Uehara-Ichiki T. (2012). Tubular structure induced by a plant virus facilitates viral spread in its vector insect. *PLoS Pathog*. 8:e1003032. 10.1371/journal.ppat.1003032 23166500 PMC3499585

[B8] Chen Q. Zhang L. Chen H. Xie L. Wei T. (2015a). Nonstructural protein Pns4 of rice dwarf virus is essential for viral infection in its insect vector. *Virol*. *J*. 12:211. 10.1186/s12985-015-0438-6 26646953 PMC4673743

[B9] Chen Q. Zhang L. Zhang Y. Mao Q. Wei T. (2017). Tubules of plant reoviruses exploit tropomodulin to regulate actin-based tubule motility in insect vector. *Sci*. *Rep*. 7:38563. 10.1038/srep38563 28067229 PMC5220352

[B10] Fukushi T. (1940). Further studies on the dwarf disease of rice plant. *J*. *Fac*. *Agr*. *Hokkaido Imperial Univ*. 45 83–154.

[B11] Gamez R. (1973). Transmission of rayado fino virus of maize (Zea mays) by *Dalbulus maidis*. *Ann. Appl*. *Biol*. 73 285–292. 10.1111/j.1744-7348.1973.tb00935.x 4701061

[B12] Gildow F. E. (1982). Coated vesicle transport of luteoviruses through salivary glands of *Myzus persicae*. *Phytopathology* 72 1289–1296. 10.1099/vir.0.19415-0 14645929

[B13] Gray S. Gildow F. E. (2003). Luteovirus-aphid interactions. *Annu. Rev. Phytopathol.* 41 539–566. 10.1146/annurev.phyto.41.012203.105815 12730400

[B14] Hattori M. Konishi H. Tamura Y. Konno K. Sogawa K. (2005). Laccase-type phenoloxidase in salivary glands and watery saliva of the green rice leafhopper, *Nephotettix cincticeps*. *J*. *Insect. Physiol*. 51 1359–1365. 10.1016/j.jinsphys.2005.08.010 16216260

[B15] Hogenhout S. A. Ammar el D. Whitfield A. E. Redinbaugh M. G. (2008). Insect vector interactions with persistently transmitted viruses. *Annu*. *Rev*. *Phytopathol.* 46 327–359. 10.1146/annurev.phyto.022508.092135 18680428

[B16] Honda K. Wei T. Hagiwara K. Higashi T. Kimura I. Akutsu K. (2007). Retention of Rice dwarf virus by descendants of pairs of viruliferous vector insects after rearing for 6 years. *Phytopathology* 97 712–716. 10.1094/PHYTO-97-6-0712 18943602

[B17] Ishikawa T. (1928). The merit of Hatsuzo Hashimoto, the earliest investigator of stunt disease of rice plant. *Nippon Plant Protect*. *Soc*. 15 218–222.

[B18] Jia D. Chen Q. Mao Q. Zhang X. Wu W. Chen H. (2018). Vector mediated transmission of persistently transmitted plant viruses. *Curr*. *Opin*. *Virol*. 28 127–132. 10.1016/j.coviro.2017.12.004 29306179

[B19] Lan H. Chen H. Liu Y. Jiang C. Mao Q. Jia D. (2016). Small interfering RNA pathway modulates initial viral infection in midgut epithelium of insect after ingestion of virus. *J*. *Virol*. 90 917–929. 10.1128/JVI.01835-15 26537672 PMC4702677

[B20] Mao Q. Liao Z. Li J. Liu Y. Wu W. Chen H. (2017). Filamentous structures induced by a phytoreovirus mediate viral release from salivary glands in its insect vector. *J*. *Virol*. 91:e00265-17. 10.1128/JVI.00265-17 28381575 PMC5446657

[B21] Miyazaki N. Wu B. Hagiwara K. Wang C. Y. Xing L. Hammar L. (2010). The functional organization of the internal components of rice dwarf virus. *J*. *Biochem*. 147 843–850. 10.1093/jb/mvq017 20190042

[B22] Muniyappa V. Venkatesh H. M. Ramappa H. K. Kulkarni R. S. Zeidan M. Tarba C. Y. (2000). Tomato leaf curl virus from Bangalore (ToLCV-Ban4): sequence comparison with Indian ToLCV isolates, detection in plants and insects, and vector relationships. *Arch*. *Virol*. 145 1583–1598. 10.1007/s007050070078 11003471

[B23] Nakagawa A. Miyazaki N. Taka J. Naitow H. Ogawa A. (2003). The atomic structure of rice dwarf virus reveals the self-assembly mechanism of component proteins. *Structure* 11 1227–1238. 10.1016/j.str.2003.08.012 14527391

[B24] Omura T. Yan J. (1999). Role of outer capsid proteins in transmission of phytoreovirus by insect vectors. *Adv*. *Virus Res*. 54 15–43. 10.1016/s0065-3527(08)60364-4 10547673

[B25] Pu L. Xie G. Ji C. Ling B. Zhang M. Xu D. (2012). Transmission characteristics of southern rice black-streaked dwarf virus by rice planthoppers. *Crop Prot*. 41 71–76. 10.1016/j.virusres.2017.11.012 29141205

[B26] Reynaud B. Peterschmitt M. (1992). A study of the mode of transmission of maize streak virus by cicadulina mbila using an enzyme-linked immunosorbent assay. *Ann*. *Appl*. *Biol*. 121 85–94. 10.1111/j.1744-7348.1992.tb03989.x

[B27] Sogawa K. (1965). Studies on the salivary glands of rice plant leafhoppers. I. Morphology and histology. *Jpn*. *J*. *Appl*. *Entomol*. *Zool*. 9 275–290. 10.1303/jjaez.9.275

[B28] Sogawa K. (1967). Studies on the salivary glands of rice plant leafhoppers. II. Origins of the structural precursors of the sheathmaterial. *Appl*. *Entomol*. *Zool*. 2 195–202. 10.1303/aez.2.195

[B29] Sogawa K. (1968). Studies on the salivary glands of rice plant leafhoppers. III. Saliva phenolase. *Appl*. *Entomol*. *Zool*. 3 13–25. 10.1303/aez.3.13

[B30] Wei T. Kikuchi A. Moriyasu Y. Suzuki N. Shimizu T. Hagiwara K. (2006a). The spread of Rice dwarf virus among cells of its insect vector exploits virus-induced tubular structures. *J*. *Virol*. 80 8593–8602. 10.1128/JVI.00537-06 16912308 PMC1563882

[B31] Wei T. Li Y. (2016). Rice Reoviruses in insect vectors. *Ann*. *Rev*. *Phytopathol*. 54 99–120. 10.1146/annurev-phyto-080615-095900 27296147

[B32] Wei T. Shimizu T. Hagiwara K. Kikuchi A. Moriyasu Y. Suzuki N. (2006b). Pns12 protein of rice dwarf virus is essential for formation of viroplasms and nucleation of viral-assembly complexes. *J*. *Gen*. *Virol*. 87 429–438. 10.1099/vir.0.81425-0 16432031

[B33] Zhong B. Kikuchi A. Moriyasu Y. Higashi T. Hagiwara K. Omura T. (2003). A minor outer capsid protein, P9, of rice dwarf virus. *Arch*. *Virol*. 148 2275–2280. 10.1007/s00705-003-0160-3 14579184

