# Supplementary material for: Viral Release Threshold in the Salivary Gland of Leafhopper Vector Mediates the Intermittent Transmission of Rice Dwarf Virus
Source: Front Microbiol. 2021 Feb 4;12:639445. doi: 10.3389/fmicb.2021.639445 (PMC7890075; doi:10.3389/fmicb.2021.639445)
Supplement: Supplementary file 1 [file Image_1.pdf]

Supporting information

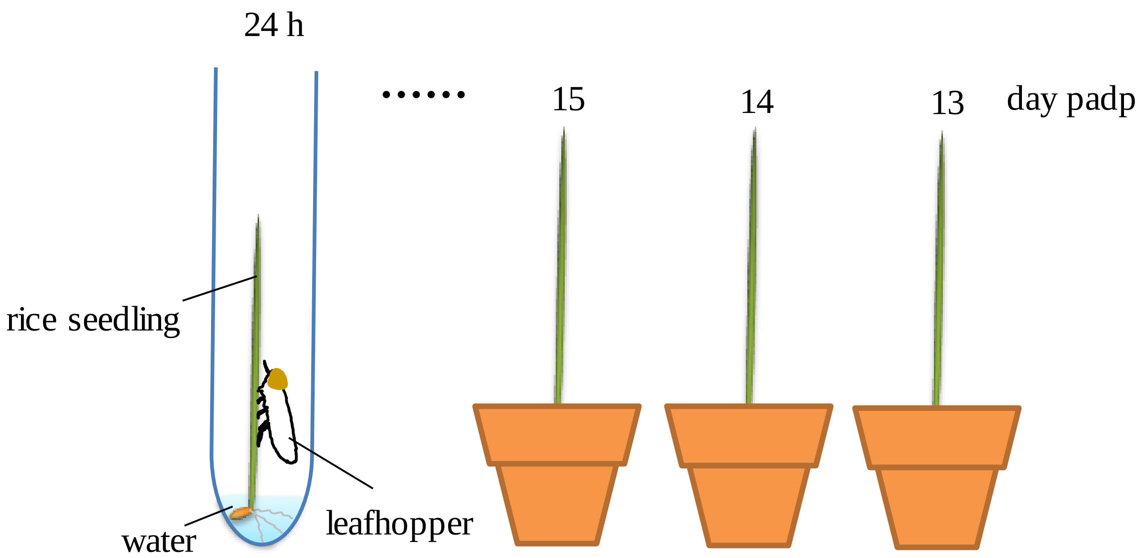

Supplementary Figure 1 Schematic illustration of viral transmission to individual rice seedings by individual leafhoppers for 13 days. Leafhoppers feeding on diseased rice plants before were individually fed on a healthy rice seedling in one glass tube for 24 h. The leafhoppers were then transferred daily to new healthy rice seedlings for 13 days. All of the tested rice seedlings were planted in an insect-proof greenhouse for 60 days.
